# Supplementary material for: Slow-Cycling Cells in Glioblastoma: A Specific Population in the Cellular Mosaic of Cancer Stem Cells
Source: Cancers (Basel). 2022 Feb 23;14(5):1126. doi: 10.3390/cancers14051126 (PMC8909138; doi:10.3390/cancers14051126)
Supplement: Supplementary file 1 [file cancers-14-01126-s001.zip › cancers-1591855-Supplementary Figures.pdf]

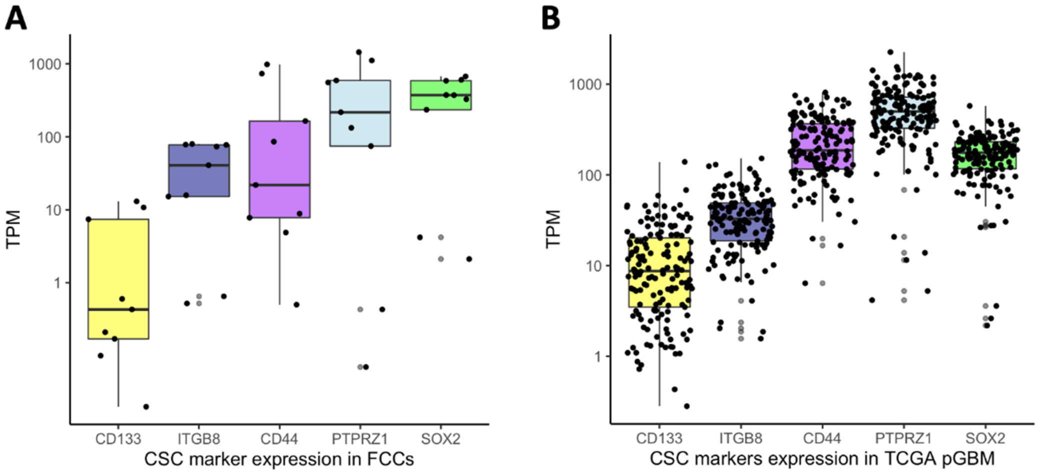

Figure S1. (related to Figure 1). Bulk RNA sequencing analysis. CSC marker expressions are represented for FCCs isolated from nine GBM patients (**A**) and for total unselected GBM cells from 153 patients (TCGA database) (**B**). Whiskers represent the 95% confidence interval and the box characterizes the interquartile range (IQR; 25th-50th-75th percentiles).

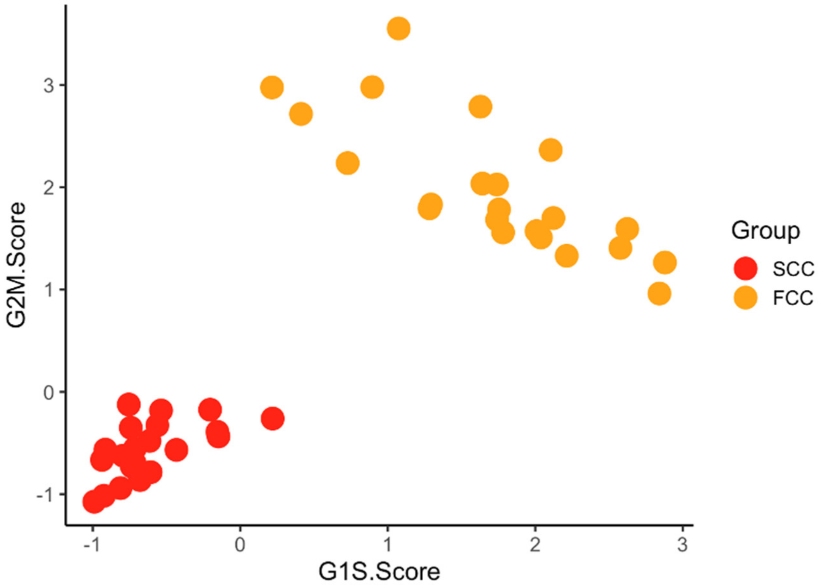

Figure S2. (related to Figure 2). Cell cycle score. Scatter plot representing the cell cycle scores of SCCs (red, n = 22) and FCCs (orange, n = 22).

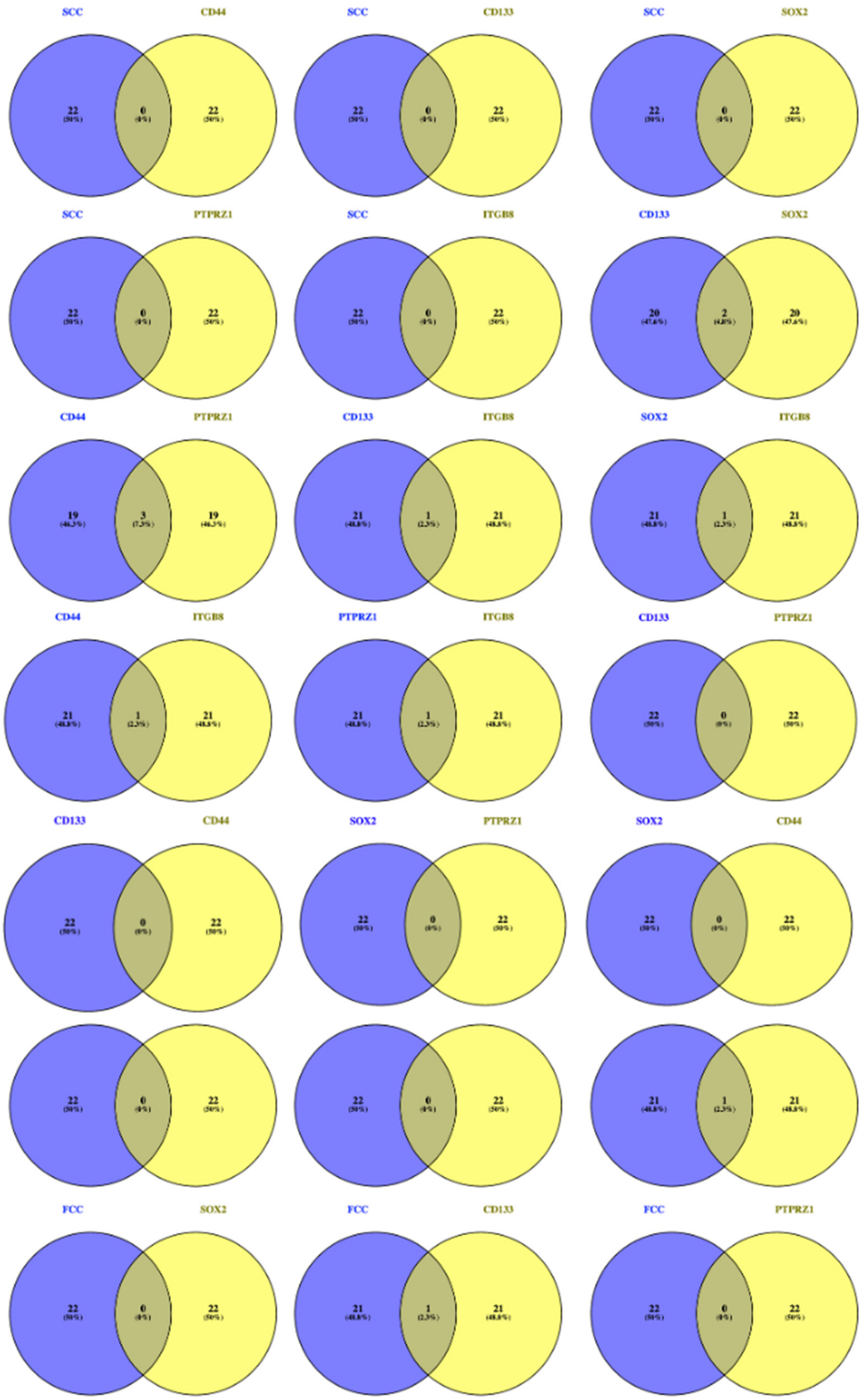

Figure S3. (related to Figure 3). Pairwise Venn Diagrams. The numbers in the overlapped areas of each Venn Diagram represent the number of cells in common between the two groups. The non-overlapping sections show the number of cells private to each population.

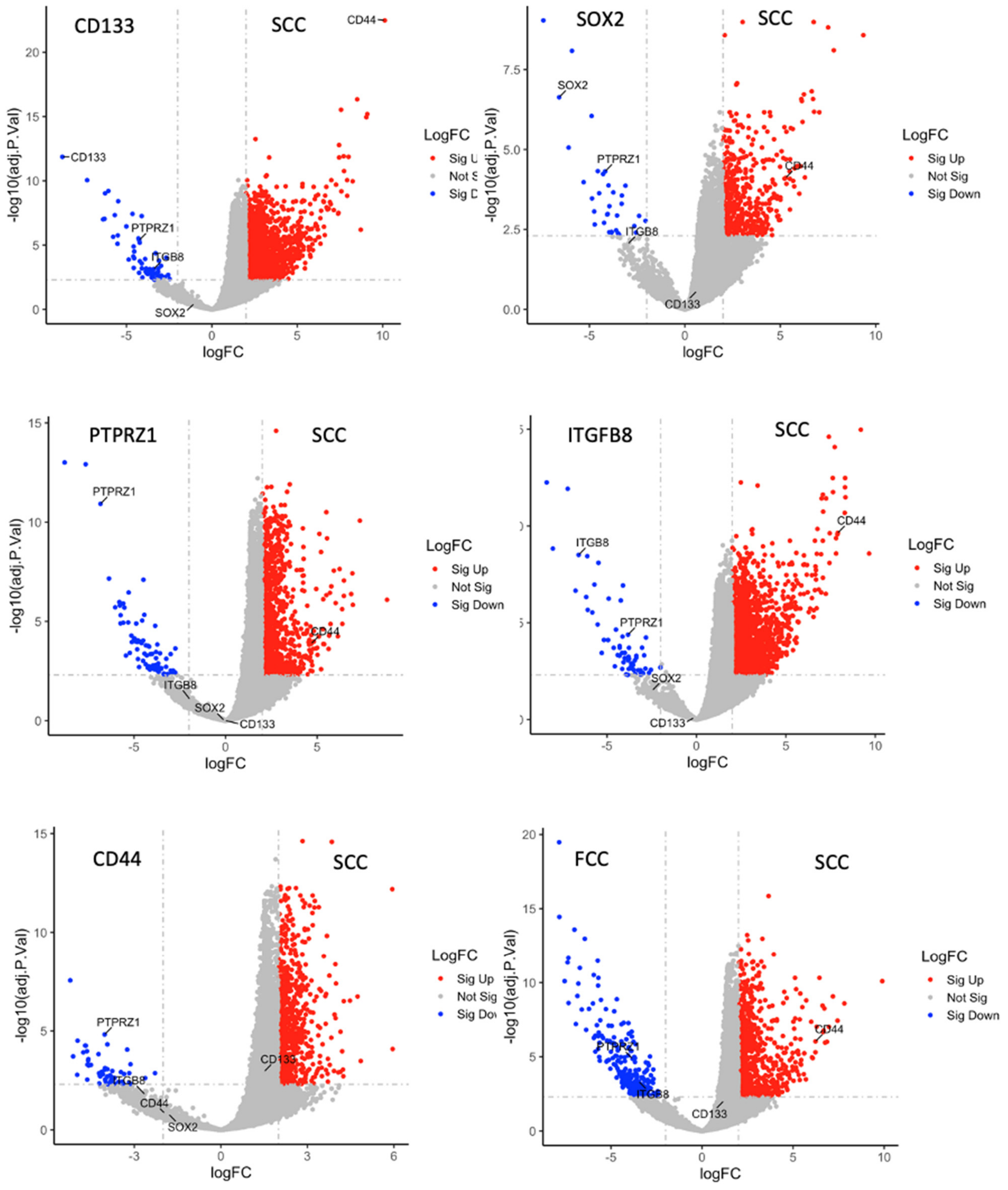

Figure S4. (related to Figure 3). Volcano plots comparing gene expression between SCC and each of the other groups. Red dots represent significantly upregulated genes in SCCs. Blue dots are significantly downregulated genes in SCCs. CSC markers (CD133, SOX2, PTPRZ1, ITGB8, CD44) were annotated. Differentially Expressed Genes (DEGs) were filtered based on LogFC over than 2 or less than -2 with an adjusted *p*-value set as 0.05.

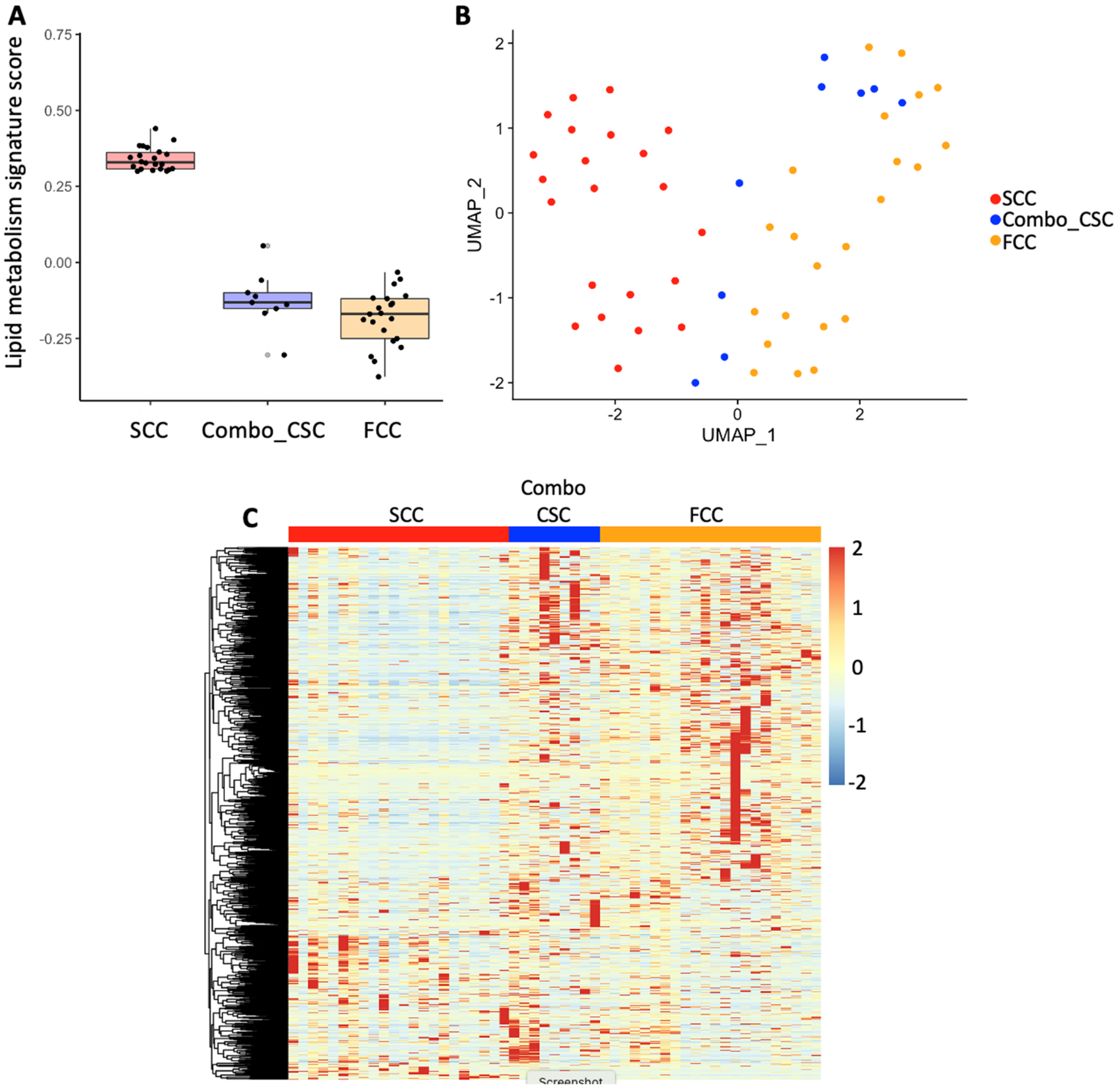

Figure S5. (related to Figure 3). (**A**) Deconvolution score of lipid metabolism signature compared between SCC, Combo_CSC, and FCC groups. (**B**) UMAP projection of scRNA-seq data comparing SCCs, Combo_CSCs, and FCCs. C) Hierarchical clustering of the top 1000 variable genes from SCCs, Combo_CSCs, and FCCs.

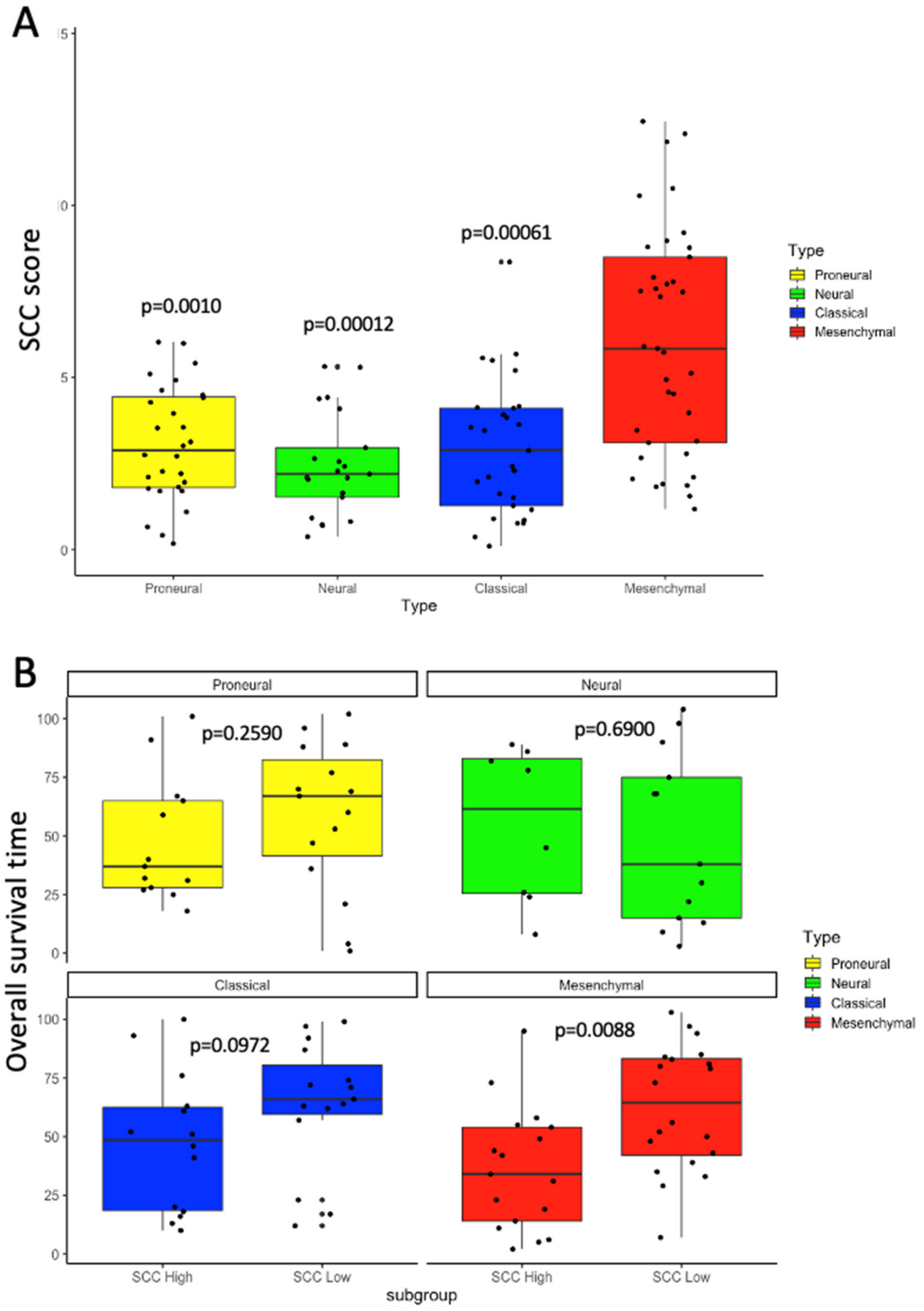

Figure S6. (related to figure 4)**.** (**A**) SCC scores between molecular subtypes. Wilcoxon test, all p-values adjusted for multiple comparisons using Bonferroni method comparing proneural, neural, and classical subgroups to the mesenchymal group. (**B**) Overall survival time (days) are compared between SCC^{high} and SCC^{low} within each molecular subtype. P values (Adjusted using Bonferroni method) of Wilcoxon test are indicated. Whiskers represent the 95% confidence interval and the box characterizes the interquartile range (IQR; 25th-50th-75th percentiles).

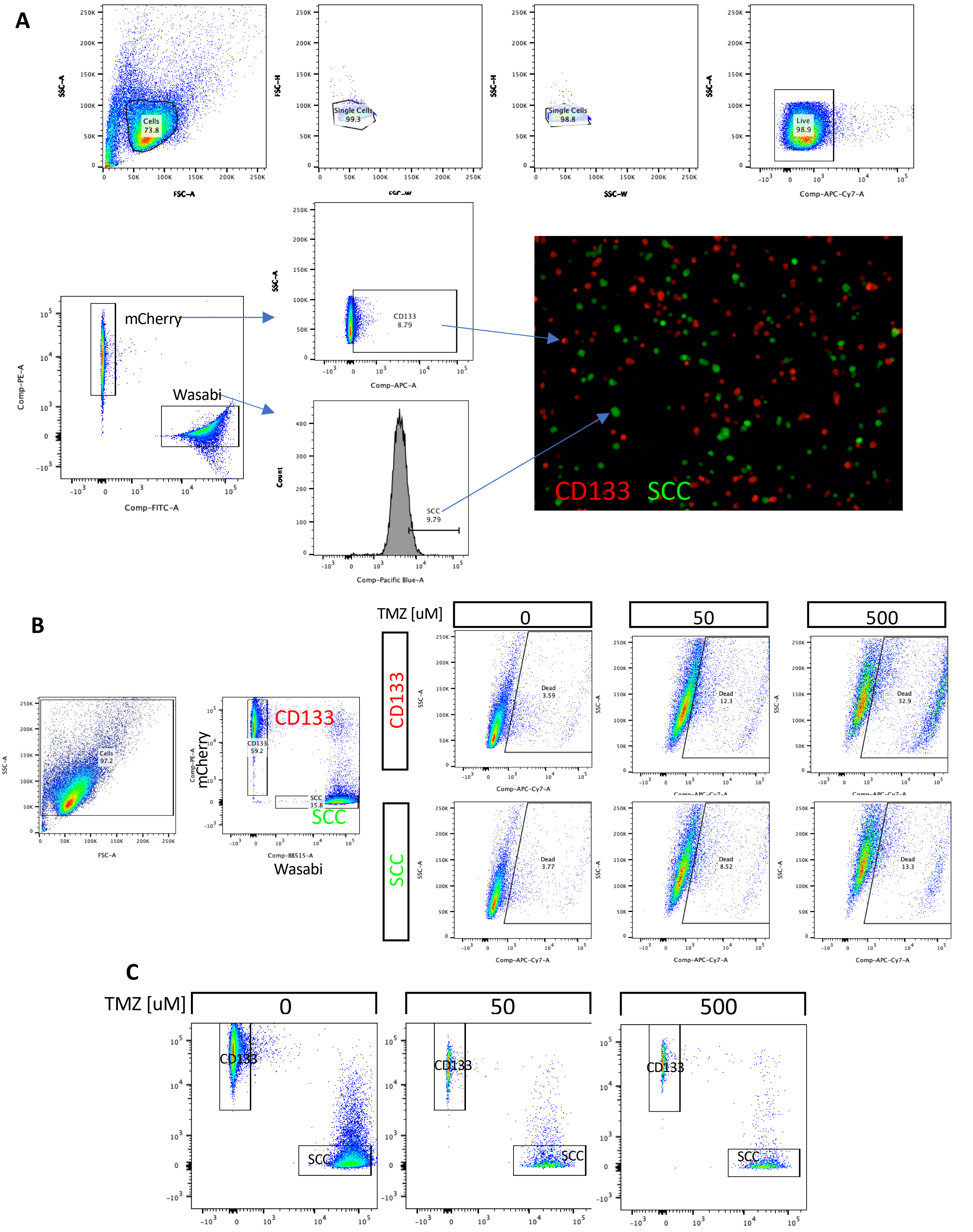

Figure S7. (related to Figure 4). Gating strategy for sorting CD133+ cells and SCCs and functional TMZ sensitivity assay. (**A**) Primary hGBM-L0 cells [5,6] were transduced to constitutively express

the fluorescent reporter tag Wasabi or mCherry. Wasabi expressing cells were labeled with CellTrace Violet and chased for one week before isolating SCCs (top 10%). mCherry-tagged CD133 immunoreactive cells were also FAC sorted and co-cultured with wasabi- tagged SCCs at an initial 40/60 ratio of SCC/CD133. The cells were then cultured and treated with various doses of TMZ. (**B**) Gating strategy to measure cell death. The percentage of dead cells was quantified by flow cytometry after incubating the cultures with live/dead fixable reactive dye. The gating strategy is presented. (**C**) The ratio of SCC/CD133 cells was evaluated by quantifying using flow cytometry the percentage of wasabi+ cells and mCherry+ cells in the different experimental groups. Representative flow dot plots are presented.

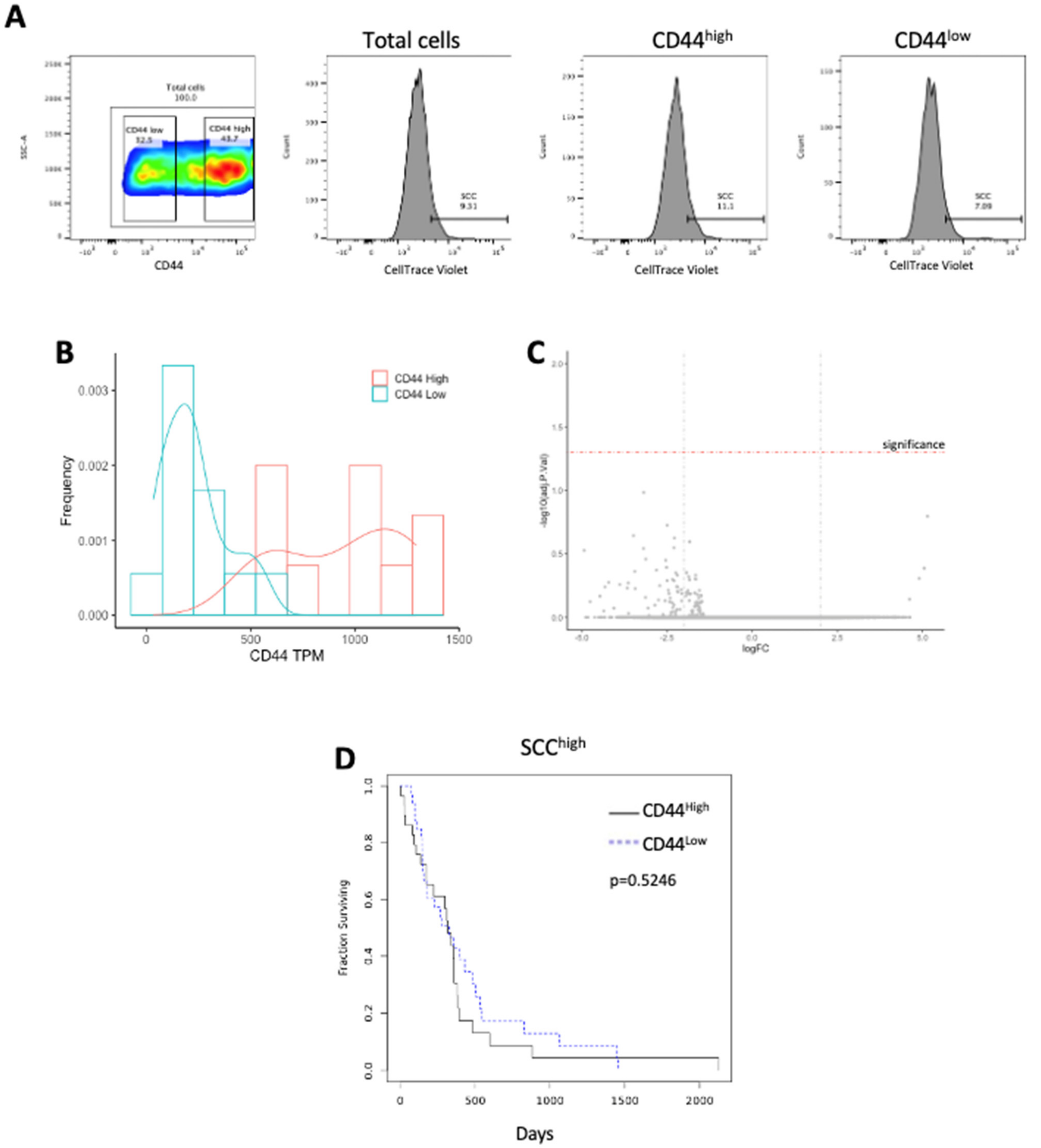

Figure S8. (**A**) Using flow cytometry, we compared the percentage of slow-cycling cells in the CD44^{high}, CD44^{low}, and total cell populations. (**B**) Single cells in SCCs (n = 22) were divided into two groups; CD44^{high} (n = 10 cells) and CD44^{low} (n = 12 cells) based on CD44 expression mean value. (**C**)

Volcano plots comparing gene expression between SCCs exhibiting high and low expression of CD44. No gene was differentially regulated between both populations, suggesting that CD44 does not drive SCC character. (**D**) GBM patients from the TCGA database with high level of SCC signature as defined in figure 4B, were split into two groups ($CD44^{high}$, $n = 29$; and $CD44^{low}$, $n = 35$). Survival time comparison showed no difference between both groups.
